# Problematic use of generative artificial intelligence chatbots: current stage of conceptual and clinical understanding

**DOI:** 10.3389/fpubh.2026.1816917

**Published:** 2026-05-28

**Authors:** Octavian Vasiliu

**Affiliations:** 1 Discipline of Psychiatry II, Department of Clinical Neurosciences, Carol Davila University of Medicine and Pharmacy, Bucharest, Romania; 2 Department of Psychiatry, Carol Davila University Emergency Central Military Hospital, Bucharest, Romania

**Keywords:** behavioral addiction, chatbots, gambling disorder, gaming disorder, generative AI, postmodernism, problematic use of the internet

## Abstract

The potential negative consequences of generative AI are the subject of intense debate, with pragmatic and theoretical arguments and counterarguments advanced by mental health specialists, researchers, and policymakers regarding its impact on creativity, overall functioning, and psychosocial wellbeing. The current narrative review addresses historical and conceptual developments in the problematic use of generative AI chatbots (PUGAIC) and empirical findings on its epidemiology, risk factors, assessment instruments, and proposed pathophysiological mechanisms, without advancing PUGAIC as a formally established diagnostic entity. Preliminary data suggest that emotional attachment, anthropomorphism, instant reinforcement, and parasocial dynamics may contribute to compulsive use of generative AI in vulnerable individuals. However, current evidence remains limited, without clinical correlates, predominantly cross-sectional, and culturally constrained. Existing measurement tools are in early stages of validation, and diagnostic boundaries between high engagement in generative AI-related activities, problematic use, and addiction-like behavior remain unclear. While moral apprehension and overpathologization are pitfalls that should be avoided, clinicians have to remain attentive to cases involving functional impairment, psychological distress, and loss of control. In conclusion, based on the available data, PUGAIC may be conceptualized as a potential spectrum phenomenon embedded within broader psychosocial vulnerabilities rather than as an established clinical disorder. Longitudinal research, cross-cultural validation of instruments, and neurocognitive investigations are needed to clarify its nosological status and inform preventive and therapeutic strategies.

## 1 Introduction

**Figure 1:**
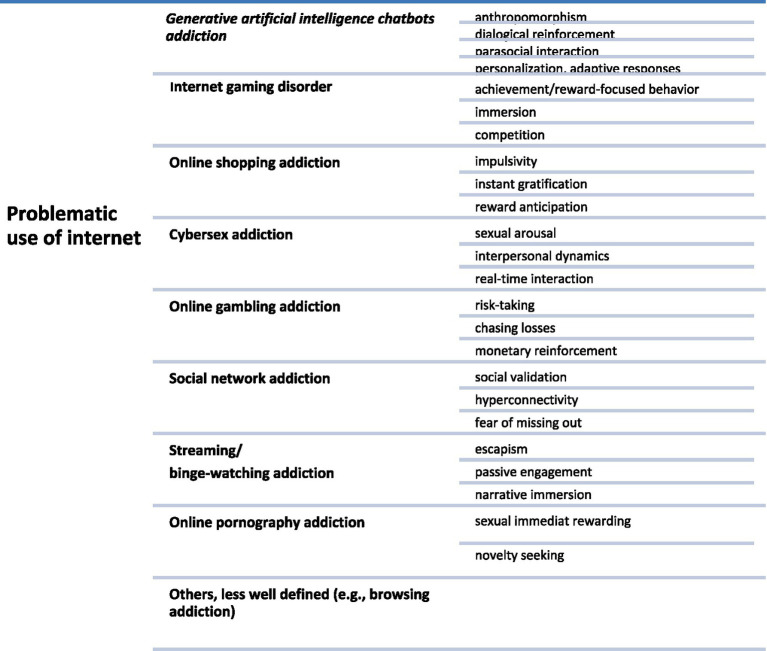
Different types of internet-related addictions.

Generative artificial intelligence chatbots (GAIC) have become increasingly popular in recent years, a trend that correlates with their expanding range of applications, from education and healthcare to software development and business and marketing. This type of expansion has raised concerns about the development of GAIC addiction, a phenomenon conceptually related to similar behavioral addictions, most closely related being problematic use of the Internet (PUI), social media addiction, or cybersexual addiction (1–4). The most recent classification systems for psychiatric diagnoses, i.e., the American Psychiatric Association’s Diagnostic and Statistical Manual of Mental Disorders, 5th edition, revised (DSM-5 TR), and the World Health Organization’s International Statistical Classification of Diseases and Related Health Problems, 11th edition (ICD-11), acknowledge the nosographic status of behavioral addictions (5, 6). DSM-5 TR recognizes the existence of Gambling Disorder and considers Internet Gaming Disorder a condition warranting further exploration, whereas ICD-11 includes Gambling Disorder and Gaming Disorder, with operationalized sets of criteria (5, 6). Therefore, interest in multiple activities related to Internet use has increased in the last decade, as they have been considered potential addictive behaviors deserving closer research. In this context, GAIC may be construed as part of an intriguing emerging direction of investigation in the contemporary psychiatric nosography. As illustrated in Figure 1, GAIC addiction may be positioned within the broader category of PUI, highlighting its relationship with other established or emerging behavioral addictions, while emphasizing its distinct place in this category of phenomena.

Placed in the center of a large category of addictive behaviors, PUI refers to the difficulty in controlling the way Internet users are able to manage their relationship with different domains of the digital world (7, 8). Noteworthy, generalisations and oversimplifications are to be avoided in this field, since some of these activities are necessary for professional life, in some cases, for example, accessing social networking on a daily basis for Public Relations specialists, while other domains are used mainly for entertainment, such as in the case of gaming, and still others are needed for usual activities, like online shopping. While trying not to overpathologize adaptive behaviors, it is also important for mental health specialists to watch carefully for dysfunctions in daily activities related to PUI and derived addictive behaviors. Also, from a psychiatric perspective, the current methods of diagnosis often fail to distinguish between problematic use of the Internet as an epiphenomenon of underlying disorders or psychological conflicts (such as anxiety, depression, personal traumas, maladaptive coping with stress, or interpersonal difficulties) and PUI as a primary disorder (7). Therefore, it seems logical and of practical interest to shift the focus away from “Internet addiction” as a standalone diagnosis and to move toward examining more specific problematic online behaviors and their psychosocial risk factors and context (7).

To avoid terminological ambiguity, especially in such a new field, several clarifications are needed. In this review, *problematic use* refers to maladaptive patterns of GAIC interaction associated with functional impairment and/or psychological distress and includes a range of limited clinical characteristics, unlike *addiction*, which implies a more severe and persistent pattern of behavior, with core criteria such as loss of control, tolerance, withdrawal, and continued use despite negative consequences. *Dependence*, as applied to GAIC, may be conceptualized as a state of psychological reliance on chatbots, characterized by frequent use and perceived need, without necessarily losing control or experiencing a well-defined functional impairment. Otherwise formulated, problematic use may be considered at the basis of a pyramid representing the severity of GAIC-associated pathological phenomenology, the next level being represented by dependence, which incorporates problematic use, and the ultimate level is the more clinically relevant aspect of addiction, including the dependence on GAIC. Since no GAIC use-related pathology is considered an established disorder within DSM-5 TR or ICD-11, this dimensional perspective is adopted by the current review because it adequately captures the complexity of GAIC use-related psychopathological phenomena. Also, such an approach may facilitate a more nuanced understanding among clinicians and researchers of their mechanisms, clinical manifestations, and implications for assessment and intervention. Regarding GAIC analysis through the lens of addictive behaviors, the core dimensions that are worthy of exploration may be classified into the historical evolution of GAIC, the benefits vs. potential harms rapport, the domains of GAIC that may be vulnerable to developing addictions, and practical considerations regarding this potential pathology (diagnostic criteria, detection strategies, risk factors, treatment options, etc.).

## 2 Methodology

This narrative review aimed to synthesize current theoretical and empirical evidence regarding the problematic use of GAIC and followed the Scale for the Assessment of Narrative Review Articles (SANRA) principles to ensure maximum methodological transparency (9). A non-systematic literature search was conducted across major electronic databases (e.g., PubMed, Scopus, Web of Science, and Google Scholar) to identify publications up to January 2026. Search terms included combinations of “generative AI,” “ChatGPT,” “chatbot addiction,” “AI dependence,” “problematic AI use,” and “technology addiction.” Peer-reviewed articles, theoretical papers, validation studies of measurement instruments, and epidemiological reports were considered. Given the emerging nature of the topic, conference proceedings and preprints, as well as sources from the grey literature, were also screened when directly relevant, i.e., when they addressed GAIC’s problematic use and provided empirical or conceptual relevance. No language restrictions were applied to the papers searched. Also, no formal quality appraisal tool was applied due to the narrative design, but priority was given to studies with clearly defined methodologies and validated instruments. The aim of this review was exploratory and integrative rather than exhaustive, seeking to outline conceptual frameworks, empirical findings, and future research directions.

## 3 Thematic overview of the literature

Out of the 154 records collected during the initial search based on keywords, only 72 publications were preserved for full-text evaluation after title and abstract screening. In the final synthesis, 48 articles were included due to their relevance and conceptual significance.

### 3.1 Historical aspects

If we consider MIT Joseph Weizenbaum’s *ELISA* as the first Chatbot created (1966), in the sense of a computer program for studying the language between man and machine, the difference from modern GAIC is significant: the first chatbots were rule-based, using pattern matching and scripted replies, but without learning or memory abilities, while GAIC is based on large language models (LLMs) and uses neural networks and probability to generate language (10, 11). GAIC began to emerge in the early 2010s, with IBM Watson (12) demonstrating machine-learning-based language understanding. Although the history of GAIC is quite short, with ChatGPT (Chat Generative Pre-Trained Transformer), the leader of this market today, being launched in 2022, the popularity of such technology has become astounding (13). Also, predictions for the chatbot market indicate expected growth to USD 11.45 billion in 2026 and to USD 21.45 billion by 2031 (14). Regarding users, recent statistics show that more than 987 million people worldwide use GAIC (15). However, the first mention of GAIC addiction in the scientific literature was an article by Zhou and Zhang (16), which was preceded only by more general concepts, such as technology addiction or Internet addiction (17–22). This situation clearly reflects a gap between the scientific advances of GAIC, the social acceptance of this technology, and the medical research in the respective field.

### 3.2 The benefits vs. harms balance

This section addresses the balance between benefits and potential harms associated with GAIC use across multiple domains, including education, healthcare, professional activities, and everyday life, rather than focusing exclusively on psychiatric applications. The term “benefits” refers to functional, cognitive, and psychosocial advantages, whereas the category of “harms” includes potential negative effects on psychological functioning, behavior, and real-world performance. However, this categorization is not absolute, since individual vulnerability factors, patterns of use, and contextual variables may make it difficult to differentiate between potential benefits and harms for a given person. In this context, it is essential to distinguish between adaptive, high-frequency use and maladaptive patterns associated with loss of control or functional impairment. Accordingly, GAIC use may be best conceptualized along a continuum ranging from adaptive engagement to problematic use, consistent with a spectrum-based perspective.

Whether used for writing homework or essays, programming code, or simply for planning travel or giving health advice, GAIC has been integrated into the daily lives of millions of users, regardless of age (13, 23, 24). Multiple applications in medicine have been proposed for GAIC, including summarizing historical patient records, reducing the time physicians spend processing data, and even serving as adjuvant psychotherapists for patients with substance use disorders (25). AI-based applications include predictive diagnostic models, screening tools, telepsychiatry, and avatar-assisted interventions across various domains (26).

Applications of artificial intelligence in psychiatry are diverse and consist of predictive modeling for diagnosis and prognosis, clinical decision-support algorithms for treatment selection, monitoring patient progress using wearable-generated data, AI-driven chatbots that deliver personalized and timely interventions, and analysis of therapy session transcripts to improve treatment quality and fidelity (27). Such interventions may be useful for increasing therapeutic adherence, monitoring patient evolution, and avoiding stigmatization by providing continuous, non-critical evaluation of the patient’s health status (28, 29).

Besides the positive effects of ChatGPT on learning, such as personalized learning, round-the-clock support, repetition and spaced learning, and interactive learning, some negative effects have been hypothesized, i.e., overreliance of users on AI, impaired critical thinking, propagation of inaccurate or misleading information, superficial engagement, reduced human interaction, and demotivation (30). For example, an MIT Media Lab study suggests that while voice-based interactions during chatbot use may initially help reduce feelings of loneliness, these benefits tend to fade, and heavier use is actually linked to increased loneliness, stronger emotional reliance on the GAIC, and less real-world social interaction (31). Importantly, it is not so much the type of conversation that matters as how often the chatbot is used, especially for individuals who are already more emotionally inclined to connect with AI (31). Another study conducted in the same interdisciplinary laboratory in Massachusetts investigated, using EEG, behavioral analysis, and essay evaluation, how reliance on LLMs affects cognitive processes during writing (32). Significantly lower brain connectivity and reduced cognitive engagement were reported among participants using ChatGPT compared with those relying on their own thinking or search engines, and greater dependence on AI was also associated with poorer memory recall, a weaker sense of authorship, and difficulty recalling one’s own work (32). It is important to note that even after participants stopped using the AI, some signs of reduced neural engagement persisted, raising concerns about long-term effects (32).

### 3.3 Domains of generative AI chatbots with addictive potential

**Figure 2:**
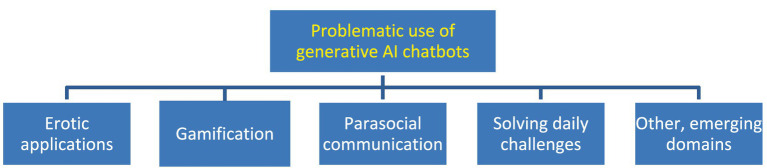
Domains of exploration for problematic use of generative AI chatbots.

The problematic use of GAIC, similar to PUI, is a heterogeneous concept, at least from a theoretical perspective. There are various domains that raise interest regarding a potential addictive property and highlight the heterogeneity of the construct, and, as shown in Figure 2, psychological, technological, and behavioral components of PUGAIC contribute to the multifaceted nature of this phenomenon.

*The erotic component of the chatbot* is one of the components that raised the question about addiction or problematic GAIC use. Replika, an app that uses AI to mimic human conversations, has removed its erotic function (i.e., Erotic Role Play, ERP) (33). Parasocial attachment was intensified by sexualized emotional bonding and a hypothesized reinforcement of the dopamine loop, presumably heightening the risk of compulsive use by blurring emotional, romantic, and sexual boundaries. An exploratory qualitative study investigated the consequences of ERP removal for user interactions on a subreddit and revealed the complex, evolving nature of human–AI interactions (33). Reactions to the removal of ERP, including grief, anger, distress, and a sense of loss, mirror withdrawal-like responses, suggesting that GAIC use can produce patterns similar to behavioral addiction, even if not clinically diagnosable (33). ERP acted as a high-intensity bonding mechanism, reinforcing attachment through sexualized and exclusive interaction. Its centrality in user narratives indicates that specific design features, rather than AI companionship per se, significantly elevate the risk of compulsive or dependent use. Many users framed their Replika relationships as responses to loneliness, trauma, or unmet relational needs. The loss of ERP destabilized these coping strategies, revealing how GAIC use can become emotionally necessary, rather than merely recreational.

As an illustration of this domain, in a 50-year-old male patient with a history of depression, but without past addictive tendencies, ChatGPT was attributed by him with a female identity, modeled according to an idol he admired, including physical features and personality (34). Through daily role-playing, the patient became immersed in imagining and creating new details about the idol, and constantly updated her personality (34). This process led to the creation of five virtual lovers, all based on the same idol, and interactions with them lasted around 2 h daily (34). His sleep and professional activity were affected by infatuation with the idol, exacerbated by constant GAIC interaction; the patient reported ruminations and considerable stress (34). The solution in this case, agreed upon by the psychiatrist, was to replace all virtual lovers with two figures resembling caring female siblings and to exclude love themes (34). The evolution was positive, and his status gradually improved over the next 10 days as a result of conscientious self-management (34).

*Gamification and engagement loops* are conceptually similar to mechanisms presumed by gaming disorder, with certain important differences. While gamification activates the same behavioral reinforcement mechanisms documented in Gaming Disorder (e.g., daily streaks, emotional rewards, regular prompts, escalation of engagement), this lastly mentioned addiction is focused on achievement, competition, and mastery, not on emotional connection and validation. Unlikely Gaming Disorder, GAIC introduces dynamics stimulating emotional dependence and parasocial attachment, not gameplay mechanics.

*The parasocial communication component of GAIC* may activate similar mechanisms to social media addiction, which is derived from its benefits in the fields of connectivity and entertainment (1). However, the perils of excessive social media use, especially among teenagers, include excessive screen time, compulsive checking, and negative impacts on real-life relationships (1, 35). The parasocial communication through GAIC can lead to the perils of excessive exposure, via variable reinforcement and engagement loops, social validation and mood regulation, escapism, and coping with stress and social anxiety. Nevertheless, while social media can offer only unpredictable feedback, GAIC provides guaranteed and immediate responses, and while social media interactions are multi-directional, GAIC communication is individual and intimate.

Also, the simple *solving of daily challenges* may encourage overdependence on GAIC, ranging from school tasks and professional duties to leisure activity planning. It is difficult to assess with accuracy the addictive potential of GAIC in this field, especially when multiple individual factors need to be considered, but further exploration could help in delineating adaptive coping from pathological behavior.

With the continuous development of GAIC, it is expected that new applications aimed at improving individuals’ daily lives and replacing automated tasks could emerge, but these may also represent a domain of interest for addictology through the lens of instant gratification and reward-based conditioning.

### 3.4 The practical aspects of generative AI chatbot problematic use

This chapter presents data on epidemiology, validated instruments for problematic use of GAIC, risk factors, pathogenesis, diagnostic criteria, and treatment options. In order to avoid confusion, it must be noted that the terminology in this chapter is preserved from the sources it was extracted from, and concepts like “dependence,” “addiction,” and “problematic use” have been defined by each group of authors cited.

### 3.5 Epidemiological data

The results of various data exploring addictive use of GAIC vary across studies, due to the heterogeneity of diagnostic criteria, population included, and instruments administered. In a two-wave cohort program (N = 5,057 and, respectively, 3,843 Chinese adolescents, mean age 13.2+/−2.55 years), 17.14% experienced AI dependence initially, and 24.19% after a semester (36).

In a survey-based study in Zimbabwe (*N* = 248 undergraduate students), 32.7% of participants demonstrated addictive patterns in their GAIC use (37). Compulsive checking (with a mean value of 18.3 daily AI interactions), failed attempts to reduce usage (65.8% reported this phenomenon), and continued reliance on AI despite recognizing negative academic consequences were reported (37). Addiction severity correlated negatively with academic metrics (37). Critical thinking atrophy, diminished writing skills development and reduced content knowledge acquisition have been observed through multiple regression analysis in GAIC addiction (37).

There are many self-reported cases of problematic use of GAIC online, but consistent epidemiological studies are scarce (38). As shown in this section, findings converge in suggesting that problematic GAIC use is relatively prevalent among younger and student populations; however, divergence emerges in reported prevalence rates due to differences in measurement tools, cultural contexts, and study designs. Therefore, more high-quality studies in this direction are expected to offer a better image of the complex phenomenon of GAIC addiction/problematic use.

### 3.6 Instruments for the measurement of GAIC problematic use

At least 7 instruments for measuring problematic use of GAIC have been identified in the literature, with only a limited number of studies supporting each of these, suggesting the research in this field is still in its early phase.

*The Problematic ChatGPT Use Scale (PCUS)* was created by Yu et al. (39) on a sample of 1,040 Taiwanese adults and proved a good factorial/construct validity, high internal consistency and test–retest reliability. Usage time and depression were positively correlated with PCUS score, and males scored higher than females (39). The Turkish version of this scale was validated by Maral et al. (40) on 391 adults, and preserves the 11-item structure of the original tool. The items are scored on a 4-point Likert-type scale, from strongly disagree (1) to strongly agree (4), and total scores can range from 11 to 55 (39, 40).

Thus, the PCUS is designed to assess maladaptive patterns of ChatGPT use, including preoccupation, loss of control, and functional impairment, and its higher total scores indicate greater severity of problematic use.

The *ChatGPT Use Scale* is another self-report, 8-item instrument designed to assess the use of ChatGPT, and was developed by Bai et al. (30). Each item is measured on a 5-point Likert-type scale, with seven items ranging from strongly disagree (1) to strongly agree, and one from never (1) to always (6) (30). Factorial analysis included academic workload, academic time pressure, sensitivity to rewards, sensitivity to quality, use of ChatGPT, memory loss, academic performance, and procrastination (30). In a correlational study using this tool, heavy workload and time pressure strongly influence the use of ChatGPT for academic tasks (30). The use of ChatGPT was likely to exacerbate tendencies toward procrastination and memory loss and to decrease academic performance (30). To prevent negative effects on learning and memory from extensive use of ChatGPT, it is recommended to engage students in critical thinking and problem-solving by assigning tasks that cannot be completed with GAIC (30).

Thus, the ChatGPT Use Scale evaluates patterns of use in academic contexts, including dimensions such as workload, time pressure, and cognitive outcomes (e.g., memory and performance), with higher scores reflecting greater reliance on ChatGPT, which has been associated with increased procrastination and reduced academic performance.

The *Dependence on Artificial Intelligence Scale (DAIS)* was developed by Morales-García et al. (41) and consists of five items and uses a 5-point Likert-type scale for responses, ranging from strongly disagree (1) to strongly agree (5), with total scores varying from 5 to 25. This instrument was validated on 528 university students, and it presents a unifactorial structure and gender invariance (41).

Therefore, DAIS may be used when assessing psychological reliance on AI systems, focusing on perceived need and habitual use. Higher scores indicate stronger dependence, reflecting increased reliance on AI for decision-making and task completion.

The *Problematic Use of Conversational Artificial Intelligence scale (PUCAI-6)* was created by Hu et al. (42) and consists of six items, each of which is rated on a 5-point Likert scale, from very rarely (1) to very often (5), and refers to preoccupation/salience, loss of control, mood regulation/escape, tolerance, withdrawal/discomfort, and conflict/functional impairment.

Therefore, the PUCAI-6 measures core addiction-like dimensions, including salience, loss of control, mood regulation, tolerance, withdrawal, and functional impairment, and higher scores indicate greater severity of problematic or addiction-like use patterns.

The *Generative Artificial Intelligence Addiction scale (GAA-3)* was developed by Zhou and Zhang (16) and has a unidimensional structure, with items assessing compulsive use/loss of control, salience/preoccupation, and functional impairment. This scale was derived from another scale, created by Cao et al. (43), dedicated to assessing *WeChat* addiction.

This scale, assessing key features of compulsive use, including salience, loss of control, and functional impairment, with higher scores reflecting stronger addiction-like tendencies in GAIC use, may be useful for researchers when a detailed evaluation focused on addiction characteristics is needed.

The *AI chatbot dependence (AICD)* scale is an 8-item instrument, created by Zhang et al. (44), and includes statements such as “If unable to use AI chatbots, I would feel anxious or uncomfortable”, scored on a Likert scale, from strongly disagree (1) to strongly agree (7). Good reliability and validity were reported about this scale.

This scale is considered capable of evaluating emotional and behavioral reliance on chatbot use, including discomfort when access is restricted; higher scores indicate greater dependence and psychological reliance on AI interactions.

Another, more complex instrument, included a scale for AI dependence with five items expressing core symptoms of smartphone addiction or problematic use of smartphones adapted to AI, and these items are scored from strongly disagree (1) to strongly agree (4), but also a scale for AI Use Motivation, with 12 items representing four dimensions-escape motivation, social motivation, entertainment motivation, and instrumental motivation, each of these item being scored similarly to the AI dependence scale (36). This instrument was developed by Huang et al. (36) and was examined in a two-wave cohort study of 3,843 adolescents.

This instrument combines a dependence scale with a multidimensional assessment of use motivations, including escape, social, entertainment, and instrumental factors. Higher scores on the dependence component indicate stronger reliance on AI, while motivation scores help contextualize underlying drivers of use.

A notable limitation in the current literature concerns the heterogeneity of instruments designed to assess problematic use of GAIC. Although multiple scales have been developed (e.g., PCUS, DAIS, PUCAI-6, GAA-3, AICD), comparative analyses of their psychometric properties remain scarce. In most cases, higher scores are interpreted as reflecting greater severity of problematic use or dependence; however, the lack of standardization across scales complicates direct comparison between studies. Few studies systematically evaluate the psychometric properties of the applied instruments, and even fewer investigate the comparative use of such instruments. Moreover, several instruments appear to adapt core criteria from established frameworks of smartphone or social media addiction, such as salience, tolerance, withdrawal, and loss of control, without sufficiently examining whether these dimensions map uniquely onto GAIC-specific behaviors. This raises the risk of conceptual tautology, whereby the construct of “GAIC addiction” may be inferred from items already modeled on pre-existing addiction paradigms. Consequently, it remains unclear whether these tools capture a genuinely distinct phenomenon or merely recontextualize broader problematic Internet use within an AI-mediated environment.

### 3.7 Risk factors

A study (*N* = 223 participants) showed that openness to using new technologies (OUNT, i.e., a personality trait reflecting willingness to engage with and adopt new digital tools) and ChatGPT self-efficacy could negatively predict the onset of ChatGPT addiction (43). The same study reported that ChatGPT addiction could negatively predict continuous intention to use ChatGPT (i.e., the likelihood of ongoing or future engagement with GAIC systems) (45). OUNT and ChatGPT self-efficacy (i.e., the perceived ability to effectively use ChatGPT to accomplish tasks) could positively predict continuous intention to use ChatGPT (45). Openness was a stronger predictor of ChatGPT addiction than ChatGPT self-efficacy (45).

Mental health problems (anxiety/depression) were associated with subsequent AI dependence, and the relationship was unidirectional (36). Escape motivation (i.e., using GAIC to avoid or regulate negative emotions such as stress, anxiety, or sadness) and social motivation (i.e., using GAIC to simulate social interaction or compensate for unmet interpersonal needs) mediated the relationship between mental health problems and AI dependence, while entertainment motivation (i.e., using GAIC for enjoyment, amusement, or passing time) and instrumental motivation (i.e., using GAIC as a tool for task completion, problem-solving, or productivity enhancement) did not (36).

Social and cultural factors have been identified as risk factors in a study in Zimbabwe, with infrastructure limitations (i.e., restricted or inconsistent access to digital resources, such as internet availability or device access) driving binge use (i.e., prolonged or excessive use within a short period, often without breaks), economic pressure increasing dependency, and institutional resource constraints (i.e., limited access to educational or professional support systems, increasing reliance on GAIC) amplifying GAI appeal (37).

Across studies, data support the existence of psychological vulnerability factors such as anxiety, depression, and maladaptive coping as key contributors. Nevertheless, there are still insufficient data regarding the relative role of individual traits versus contextual and sociocultural influences.

### 3.8 Pathogenesis of PUGAIC

According to a study (*N* = 364 GAIC users), emotional attachment has been found to be positively associated with technology addiction, while functional attachment was not correlated with this dependence (46). Also, perceived anthropomorphism and perceived empathy have been reported to be associated with increased emotional attachment, while system quality and information quality had a positive effect on functional attachment (46).

An exploratory analysis of how ChatGPT can foster dependency suggests that personalized responses, emotional validation, and continuous engagement have been proposed as potential factors mediating this relationship (47). Instant gratification and adaptive dialogue may contribute to a perceived blurring of the boundary between AI and human interaction, mimicking a real social communication (47). A potential decrease in critical thinking among GAIC users has been suggested to be associated with enhanced productivity and secondary over-reliance on AI (47). Therefore, compulsive patterns of GAIC may contribute to increased tolerance and impairment of daily living (47).

Similar to smart home assistants (SHAs), such as Amazon’s Echo or Google’s Home, which are activated by voice commands and integrated with AI, the gratification theory may explain PUGAIC (48). Different types of SHA lead to varying degrees of gratification across groups, and gratification has been found to be associated with continued use intention and may contribute to addictive patterns (48). SHA may offer utilitarian, hedonic, convenience, and social or psychological gratification, reinforcing their use, according to the “use and gratification theory” (48, 49).

The “dual parasocial model” refers to the creation of a virtual relationship with a media figure using two distinct channels—the traditional parasocial interaction and the bidirectional one, involving AI-simulated figures, based on the same real individual (34). This model is intended to explain the dynamics of GAIC users who create duplicate, virtual characters based on real persons and then become infatuated with both the real and imagined characters (34). This type of dual interaction may be highly reinforcing and has been hypothesized to increase the risk of addiction-like behaviors (34). The progress of GAIC allows for the creation of a ChatGPT persona with a high level of anthropomorphism, which may enhance perceived intimacy and potentially increase the risk of dependence (34).

A study (*N* = 2,602 ChatGPT users in Vietnam) confirmed that compulsive GAIC usage has been reported to correlate with heightened anxiety, burnout, and sleep disturbance (50). Also, compulsive ChatGPT usage indirectly contributed to sleep disturbance through the mediation of anxiety and burnout (50).

An analysis of continuance intention (CI) among 370 chatbot users showed that perceived enjoyment, perceived usefulness, and perceived ease of use were identified as significant predictors of CI in this study (51). Another study (*N* = 262 students at King Faisal University in Saudi Arabia) indicated that interactive and collaborative learning enhances the chances of ChatGPT adoption by users (52). Social interaction was found to be important in this context because engaging in conversations and knowledge-sharing appeared to increase comfort while using ChatGPT; information quality influenced substantially continuing use of chatbots by researchers, while perceived ease of use and perceived usefulness played an intermediary role (50). Also, user-friendly interfaces and perceived utility were essential factors for influencing overall satisfaction levels of ChatGPT users (52).

The “self-medication” theory applied to problematic use of AI suggests that individuals will engage in the addictive behavior whenever they are faced with powerful, negative emotions, such as anxiety and depression (36, 53). Also, the compensatory internet theory implies that going online is a way to escape from real-life problems or mitigate dysphoria, thus creating a favorable terrain for the development of an addiction (54).

Four “dark patterns” of GAIC addiction potentially related to dopamine-mediated reward circuitry were hypothesized, after conducting a literature review: (a) non-deterministic responses, (b) immediate and visual presentation of responses, (c) notifications, and (d) empathetic and agreeable responses (38). The first pattern derives from the fact that GAIC technology produces responses that cannot be completely predictable, leading to reward uncertainty in users, and an increase in dopamine release; also, the “almost satisfactory” responses that GAIC can occasionally offer are similar to near-miss answers, which can temporarily boost dopamine levels (38). The second pattern is based on the possibility that chatbots include visual presentations in their answers, which are strong visual cues that may, over time, become reward-predicting cues; also, the responses are nearly instant, so that the gratification occurs immediately (38). The third pattern refers to GAIC that can initiate conversations (e.g., Character.AI), and the users will receive notifications through email; these notifications signal potentially rewarding responses, and in time become reward-predicting cues, triggering dopamine release (38). Lastly, the empathetic and agreeable responses are part of the strategy that GAIC uses for simulating genuine conversations, and on many occasions, these responses are what the user wants to hear (38).

In conclusion, reinforcement processes, emotional attachment, and anthropomorphic features are widely recognized as important factors in sustaining GAIC use. Still, the strength of the evidence varies, with some findings being empirically supported and others remaining largely theoretical or speculative.

### 3.9 Diagnosis of problematic use of GAIC and controversies regarding this concept

“GAIC addiction” is a debatable concept, and there are authors questioning the validity of such addictive potential, corresponding scales for ChatGPT addiction, and “moral panics” surrounding new technologies, in general (55). This critique highlights the risk of overpathologization and the lack of convincing negative consequences, impaired control, psychological distress, and functional impairment associated with GAIC use (55). Other authors have mentioned “public anxiety about AI” as the primary driver of the creation of PUGAIC, but, at the same time, signaled that the warning is not just hypothetical (47).

While the distinction between “problematic use of GAIC” and “GAIC addiction/dependence” is still blurry, the two concepts may be seen as different levels of the same problem, on a continuum with the non-pathological use of GAIC. The limitations regarding clear-cut diagnostic criteria may be considered normal given the current state of knowledge, since the phenomenon under investigation is still new. An evolution in this field may be expected, similar to the one observed in the domain of other behavioral addictions, some of which are currently mentioned in the DSM-5 TR and ICD-11.

However, a provisional framework may be considered to guide clinical and research perspectives, even though it is only tentative, and empirical support is strongly needed. PUGAIC may be identified when several core features are present, including (1) *loss of control over use*, reflected in unsuccessful attempts to reduce or regulate engagement; (2) *functional impairment*, such as negative effects on academic, occupational, or social functioning; (3) *emotional reliance on GAIC*, characterized by the use of these systems for mood regulation or coping with distress; and (4) *persistence of use despite awareness of negative consequences*. This framework does not imply the existence of a distinct clinical disorder; rather, it offers a preliminary structure for identifying maladaptive patterns of use within a spectrum-based conceptualization.

### 3.10 Therapeutic options

No clearly formulated and evidence-based therapeutic strategy has been found in the literature for this addictive behavior. Recommendations similar to those for Social Network Sites (SNS) and, more generally, for Internet addiction, such as integrating sociotechnological safety measures into the design of digital media to avoid fear of missing out or to limit exposure to the GAIC, can be safely extrapolated to problematic use of GAIC (56–59). Behavioral interventions, cognitive-behavioral therapy and mindfulness training, educational initiatives that raise awareness about addiction risks have been explored in SNS addiction, as well as parental strategies emphasizing boundaries and monitoring (57).

Regarding the potential therapeutic strategies, findings suggest that interventions derived from established approaches to behavioral addictions (e.g., cognitive-behavioral strategies) may be applicable, but divergence emerges due to the lack of GAIC-specific therapeutic models and empirical validation.

## 4 Discussion

The emergence of problematic use of GAIC must be approached with conceptual caution because, while parallels with established behavioral addictions such as Gambling Disorder or Gaming Disorder may appear compelling at a phenomenological level, current evidence remains insufficient to support a fully operationalized diagnostic construct. Therefore, rather than fall into the pitfall of overpathologizing, it may be more appropriate to conceptualize problematic use of GAIC as a spectrum of maladaptive interaction patterns developed on a broader psychosocial vulnerability that remains to be defined by further studies.

In a broader sense, PUGAIC may be considered a pathological aspect of the human–computer interaction (HCI), with users tending to attribute social and human-like qualities to technological systems, even when they are aware of their artificial nature, thus contributing to increased engagement and perceived relational closeness (27, 60–62). Internet-use disorders were examined through the lens of HCI, and several models were proposed, including the I-PACE (Person-Affect-Cognition-Execution) (61). This process model was created to explain the development and maintenance of addictive use of Internet applications or sites dedicated to gaming, gambling, pornography viewing, shopping, or communication (61). I-PACE frames the Internet-use disorders as a consequence of interactions between vulnerability factors (e.g., neurobiological and psychological specifics), moderators (e.g., coping styles and cognitive biases), and mediators (e.g., affective and cognitive responses to situational triggers, reduced executive functioning) (61). This HCI model may also be applied to PUGAIC, since all its core elements (i.e., vulnerability, moderators, and mediators) may also be identified in the interaction between users and GAIC.

Another important aspect of technological addictions, PUGAIC included, refers to AI ethics. In this domain, literature highlights concerns about transparency, user autonomy, and the potential for manipulation by highly personalized and adaptive systems, which may lead to excessive or maladaptive patterns of use (63–65). AI systems should be designed and deployed to promote human wellbeing, preserve autonomy, and minimize harm, particularly in contexts involving vulnerable users (63). Algorithmic systems can influence human behavior in subtle and often non-transparent ways, raising concerns about transparency, accountability, and the potential for unintended bias or manipulation (64). Trustworthy AI should respect human agency and autonomy while avoiding mechanisms that may exploit users’ cognitive or emotional vulnerabilities (65). All these aspects are important when discussing PUGAIC, since anthropomorphism and parasocial interaction may be manipulated by GAIC manufacturers, as previously illustrated in the case of ERP.

Also, from a digital psychology perspective, problematic technology use has been conceptualized as a maladaptive coping strategy, in which individuals engage with digital environments to regulate negative emotions or compensate for unmet psychological needs (54, 66, 67). This aspect was mentioned in the section on pathogenesis theories of PUGAIC (59), but there are multiple dimensions worth exploring when analyzing compensatory behavior. For example, problematic GAIC use may represent a maladaptive coping strategy, whereby individuals engage with AI systems to regulate negative affect or compensate for unmet psychological needs, rather than a primary addictive disorder (66). Consistent with recent critiques of overly broad constructs such as “Internet addiction,” it has been argued that research should focus on specific behaviors rather than the technological medium itself, supporting the examination of GAIC use as a distinct behavioral pattern (67). These perspectives suggest that PUGAIC should be interpreted with conceptual caution: on the one hand, it may reflect a compensatory mechanism for managing psychological distress, rather than a primary addictive disorder, while on the other hand, consistent with recent efforts to refine the taxonomy of behavioral addictions, GAIC-related behaviors should be examined as specific interaction patterns rather than subsumed under broad and potentially misleading categories such as “Internet addiction.”

A central challenge lies in distinguishing adaptive instrumental use from compulsive, emotionally driven behavior. Unlike traditional Internet-related addictions, GAIC platforms provide personalized, immediate, and anthropomorphized interaction, potentially intensifying emotional attachment and reinforcing parasocial dynamics. For example, Gaming Disorder is an achievement-based addiction, SNS dependence is focused on social validation, Gambling Disorder has as its core features financial gains and chasing losses, for online pornography addiction, the central element is loss of control over sexual behavior, and for online shopping disorder, the behavior is modeled by the excessive preoccupation with shopping, browsing products, and planning purchases. Therefore, GAIC use may be safely differentiated from other behavioral addictions because its reinforcement is dialogical, individualized, and algorithmically optimized, which may enhance perceived intimacy and psychological salience. One of the most relevant PUGAIC characteristics is the high degree of personalization, whereby responses are dynamically adapted to the user’s preferences, behavior, and interaction history. This level of individualized feedback may enhance perceived relevance and engagement, thereby reinforcing continued use in ways that differ from those in more static digital environments.

In addition, GAIC systems are characterized by a pronounced degree of anthropomorphism, as users tend to attribute human-like qualities such as intentionality, empathy, and agency to conversational agents. This process may increase emotional involvement and foster a sense of relational closeness, which is less prominent in other forms of digital engagement, such as gaming or passive content consumption.

Furthermore, the dialogical nature of GAIC interaction represents a distinct reinforcement mechanism. Unlike social media or gaming platforms, where feedback is intermittent and externally mediated, GAIC provides immediate, continuous, and personalized responses, creating a form of dialogical reinforcement that may sustain engagement through perceived reciprocity and responsiveness. Together, these features suggest that GAIC use may involve unique interactional dynamics that extend beyond traditional models of behavioral addiction.

The current literature on problematic GAIC use presents several limitations that should be taken into account when interpreting the findings. Many studies rely on cross-sectional designs, which make it difficult to draw causal conclusions. In addition, samples are often relatively small and frequently drawn from student populations, which may limit the generalizability of the results. Another important issue is the reliance on self-report measures, which can introduce biases such as social desirability bias or recall inaccuracies. Also, given the field’s novelty, publication bias cannot be ruled out, as studies reporting significant or novel results may be more likely to be published. No less important is the fact that current epidemiological findings are preliminary and culturally bounded. Due to all these aspects, reported prevalence rates vary significantly, and validated instruments remain in early stages of development. For example, studies conducted in China and Zimbabwe differ not only in reported prevalence rates but also in sampling strategies, population characteristics, and assessment instruments (37, 45). The Chinese cohort study, based on a large adolescent sample and longitudinal design, allows for a more dynamic assessment of AI-related dependence, whereas the Zimbabwean study relies on a smaller, cross-sectional university sample, limiting generalizability (37, 45). Cultural factors may affect prevalence data through differences in educational systems, digital infrastructure, and social norms surrounding technology use, which can shape both usage patterns and perceptions of problematic behavior. Additionally, the use of heterogeneous measurement tools further complicates direct comparisons, as these instruments vary in their conceptual definitions and thresholds for problematic use. Overall, these limitations suggest that more robust, longitudinal, and cross-culturally sensitive research is needed to better understand this emerging phenomenon.

Moreover, the heterogeneity of motivations, such as escape, social compensation, productivity enhancement, and curiosity, suggests that problematic use may represent a secondary coping mechanism rather than a primary addictive disorder in many cases.

This review supports a research agenda focused on: (1) longitudinal designs clarifying causal directionality; (2) cross-cultural validation of measurement tools; (3) differentiation between primary addiction-like patterns and secondary maladaptive coping; (4) neurocognitive and affective correlates of excessive GAIC engagement.

In this stage of development, conceptual precision and methodological rigor are essential to avoid both technological moral panic and premature diagnostic inflation. As a preliminary model, a multi-stratified approach to the problematic use of GAIC may be considered, with the assessment of the following components: (A) individual risk factors (e.g., coping styles, anxiety, depression); (B) technological risk factors (e.g., immediate response triggering immediate satisfaction, anthropomorphization, simulated empathy); (C) relational processes (e.g., parasocial communication, decreased cognitive effort for the users); (D) behavioral consequences (e.g., increased time of GAIC use, tolerance, withdrawal phenomena, reduced performance in daily activities); (E) psychological addiction and negative reinforcement (e.g., decreased level of anxiety and depression).

This five-component framework was derived from a synthesis of existing models of behavioral addiction and emerging evidence specific to GAIC use. Classical addiction frameworks emphasize dimensions such as loss of control, salience, tolerance, withdrawal, and functional impairment, which have been widely applied across various forms of problematic technology use. However, these models do not fully capture the relational and interactive characteristics specific to GAIC.

Accordingly, the present model integrates both established addiction-related components and GAIC-specific features, including technological characteristics (e.g., personalization and response immediacy) and relational processes (e.g., parasocial interaction and emotional attachment). This integrative approach allows for a more nuanced understanding of GAIC-related behaviors, acknowledging both their continuity with existing addictive patterns and their distinctive interactional properties.

Importantly, the five-component structure should not be interpreted as a definitive diagnostic model, but rather as a preliminary conceptual framework intended to organize current evidence and guide future research. By combining insights from behavioral addiction theory, human–computer interaction, and digital psychology, this model aims to capture the multidimensional nature of GAIC use while remaining flexible to further empirical validation.

Taken together, these considerations support the view that GAIC use occupies a unique position within the spectrum of problematic technology use. While sharing core features with established behavioral addictions, its highly interactive, personalized, and anthropomorphized nature introduces additional mechanisms that may intensify engagement and complicate its clinical interpretation. This justifies the need for conceptual models that move beyond traditional frameworks and incorporate GAIC-specific dynamics.

## 5 Limitations and future directions

Several limitations must be acknowledged. First, this review is narrative in nature and does not follow a systematic search protocol, which may limit reproducibility and introduce selection bias. Second, the empirical literature on problematic use of generative AI chatbots remains scarce, heterogeneous, and predominantly cross-sectional, limiting the possibility of causal inferences. Third, most available data originate from adolescent or university samples and specific cultural contexts, limiting generalizability to broader populations. Fourth, measurement instruments assessing GAIC dependence are newly developed and partially adapted from existing Internet or smartphone addiction scales, raising concerns about construct validity and conceptual overlap. Finally, the rapid evolution of AI technologies may outpace current research frameworks, meaning that theoretical interpretations presented here should be considered provisional and subject to revision as more robust longitudinal and neurocognitive data become available.

Due to the early stage of research on problematic use of GAIC, future studies should prioritize longitudinal designs to clarify causal relationships between GAIC engagement and mental health outcomes. Cross-cultural validation of newly developed assessment tools is essential to strengthen construct validity and differentiate between high engagement and clinically relevant impairment. Neurocognitive and affective mechanisms underlying excessive GAIC use—particularly reward processing, cognitive offloading, and emotional attachment—require systematic investigation. Further research should also distinguish functional, productivity-oriented use from emotionally driven reliance and parasocial attachment. Design-oriented and clinical studies are equally important. Exploring how interface features may reinforce compulsive patterns, as well as testing targeted preventive and therapeutic interventions, will be crucial. At this stage, conceptual refinement and methodological rigor remain necessary to avoid both diagnostic inflation and under-recognition of clinically significant cases.

## 6 Conclusion

The emergence of GAIC marks an evolution in technology that can significantly impact the development of human knowledge. In postmodern society, facing specific challenges in perceptions of autonomy and behavioral self-regulation, the role of generative AI remains a matter of debate. From the perspective of mental health specialists, the problems raised by GAIC are similar to other behavioral addictions, some of which gained new nuances with the onset of the Internet (e.g., gaming, shopping, or pornography addictions), while others are completely new (e.g., SNS addiction). While several authors argue that AI dependence is not a cause for panic in current circumstances (35), mental health specialists should prepare to manage such cases. Significantly more data are needed on the epidemiology, diagnostic criteria, pathophysiology, and treatment of problematic use of GAIC, so that preventive policies and therapeutic strategies can be developed.
